# Memories of Dr. Walter Werner Holland

**DOI:** 10.2188/jea.JE20180157

**Published:** 2018-09-05

**Authors:** Kunio Aoki, Hiroshi Yanagawa, Kazunori Kodama, Tsutomu Hashimoto

**Affiliations:** 1 Professor Emeritus, Nagoya University; 2 Professor Emeritus, Jichi Medical University; 3 Executive Director, Radiation Effects Research Foundation; 4 Professor Emeritus, Wakayama Medical University

Dr. Walter Werner Holland

Professor Emeritus of St Thomas’ Hospital Medical School in London

Commander of the British Empire (CBE)

Fellow of the Royal College of Physicians of London (FRCP)

Fellow of the Faculty of Public Health Medicine of the Royal Colleges of Physicians of the United Kingdom (FFPH)

Professor of Health, London School of Economics and Political science (LES)

Honorary Member of the International Epidemiological Association (IEA)

We are sunk in grief after hearing that Dr. Walter Werner Holland, a distinguished leader in Epidemiology and Public Health, is with us no more. We lost an irreplaceable person in the world. Please accept our sincere condolences, and may his soul rest in peace.

Dr. Holland, who served on the IEA Executive Committee for long time, had contributed to promote the activities of Japanese epidemiologists by inviting some of them to be members of the IEA Executive Committee, by participating in international collaborative works, and by holding the international scientific meeting of the IEA (Regional and international) in Japan. He frequently delivered lectures and gave much valuable advice in Japan during his very busy days. It should be noted that he kindly accepted the role of co-planner of the postgraduate education course of Epidemiology and Public Health Medicine in Japan, responding to a request from the late Professor Itsuzo Shigematsu (Councilor of the IEA 1981–84, Former Chairman of the Radiation Effects Research Foundation [RERF], Former Head of the Department of Epidemiology of National Institute of Public Health [NIPH], Japan). Dr. Shigematsu highly appreciated the research and its practical applications to service in the local community made by Dr. Holland and his colleagues and wished to share the details with younger colleagues.

With the cooperation of the British Council in Japan, the Japan-Britain co-sponsored “British Epidemiology and Public Health Courses” was held for 1 week in January 1994 (Organizer: H. Yanagawa, Jichi Medical University), with the main theme of “The Methods and Applications of Epidemiology and Public Health”. This course was sponsored by the British Council, the Cooperative Foundation against Environmental Pollution, and the Daiwa Anglo-Japanese Foundation. It was also supported by the Japan Epidemiological Association (JEA) and the Japan Public Health Association. Dr. Holland played major roles in the course, together with his colleagues from the United Kingdom. The course was successfully completed. Japanese participants, having a great shock, wished to have another course someday. With Dr. Holland’s cooperation, the 2nd course was held in Osaka in 1996 (Organizer: Dr. T. Hashimoto, Wakayama University), followed by a 3^rd^ course in Hiroshima in 1998 (Organizer: Dr. K. Kodama, RERF). The final seminar was held in Fukuoka in 2001 (Organizer: Dr. S. Matsuda, University of Occupational and Environmental Health). We should express our thanks again to Dr. Holland for his tremendous efforts and generosity. Not only the participants in the course, but also many members of the JEA, were subsequently stimulated by the course to modify their ways to study and practice. As a consequence, the activities of JEA changed and improved.

Dr. Holland gave a presentation on the history of the IEA from 1984–1995 at the 14^th^ International Scientific Meeting (ISM) of IEA, held in Nagoya, Japan (President: Dr. K. Aoki) and introduced a book edited by him entitled “Foundations for Health Improvement: Productive Epidemiological Public Health Research 1919–1998, the Nuffield Trust, 2003”, describing the evolution of Epidemiology and Public Health Medicine in the United Kingdom and the United States in the 20th century. The book opened our eyes for developmental history. Another book, entitled “Development of Modern Epidemiology: personal reports from those who were there”, gave us a wealth of knowledge on the history of epidemiological concepts, methods, development of epidemiology by specific disease, application to public health services, and occupational and social epidemiology. The book also provided a historical review of epidemiological studies in various countries.

Now, we do not know how to express our thanks to him.

A brief profile of Dr. Walter Werner Holland is as follows:

He graduated from London St. Thomas’ Hospital Medical School in 1954, receiving medals in physiology and gynecology and scholarship. He was posted to the Central Public Health Laboratory Service of Royal Air Force, where he studied vaccines and had a chance to study influenza at the time of 1957 epidemic. Such experience stimulated him to apply for the Medical Research Council Fellowship, where Dr. Austin Bradford Hill, medical statistician, and Dr. Donald Reid, epidemiologist, were the leaders. Then, he studied in the Department of Epidemiology at Johns Hopkins University in Baltimore, Maryland, United States, for 2 years. In 1962, he became a senior lecturer in social medicine at St. Thomas’ Hospital Medical School, where he set up a new Social Medicine and Health Services Research Unit within the Department of Health in 1964. It was the first department in the United Kingdom for opening the roads in Epidemiology and Public Health Medicine, where not only researchers, physicians, medical officers, and politicians, but also laymen, worked together. Cost-benefit analysis on medical and public health projects were conducted based on scientific evidence. He served as professor and chairman of the department from 1964 to 1994.

His research career in specific areas was on chronic bronchitis, air pollution, and hypertension. He also evaluated mass screening programs of chronic diseases in multiphases, including cost-benefit analysis. He served as an important member of various national and international research and health committees, including Expert Committees of the WHO. (He was born on March 5, 1929 and died on February 9, 2018 from prostate cancer.)

Note: They say that his life in the school days was rigorous, because he immigrated to England at 10 years old. But he overcame the difficulties by his distinguished capability. His gentle and warm smile often consoled many people, and it might be based on his intelligence. It may also be due to the happy days of his childhood. In 1998, one of the joint describers, Aoki, spoke at the last lecture of Japan-British Epidemiology Course, reminiscing on “the happy dreamy days of the young”. It was just a rough recollection for the old: “in the midst of the younger days, we were always like stray sheep”. There was a popular song at the time: “so, you should go your own way having the courage”. After this lecture, Dr. Holland whispered that the remark was really good, with a gentle smile as usual. Now, we recognize how he surmounted the hardship of his youth. He was really a great man.

